# Editorial: User-centered technology for exercise optimization in older adults

**DOI:** 10.3389/fspor.2025.1558979

**Published:** 2025-02-04

**Authors:** Rafael A. Bernardes, Eleftheria Giannouli, Hugo Neves, Vítor Parola

**Affiliations:** ^1^ Centre for Interdisciplinary Research in Health (CIIS), Faculty of Health Sciences and Nursing (FCSE), Universidade Católica Portuguesa (UCP), Lisbon, Portugal; ^2^ Department of Health Sciences and Technology, Institute of Human Movement Sciences and Sport, ETH Zurich, Zurich, Switzerland; ^3^ The Health Sciences Research Unit: Nursing (UICISA:E), Nursing School of Coimbra (ESEnfC), Coimbra, Portugal

**Keywords:** older adult, exercise, physical activity, technology - assistive/supportive, artificial intelligence, user-centered design

**Editorial on the Research Topic**
User-centered technology for exercise optimization in older adults

## Introduction

The global demographic shift toward an aging population underscores the urgent need for innovative strategies to enhance health and well-being among older adults. Physical activity is a fundamental to healthy aging, reducing the risk of chronic conditions, improving mental health, and fostering independence. However, traditional approaches to exercise often fail to address the unique needs and barriers older adults face. The WHO's Global Action Plan on Physical Activity 2018–2030 (1) emphasizes the necessity of reducing physical inactivity by creating inclusive environments and implementing policies that encourage active lifestyles across all age groups. For older adults, the report highlights the importance of accessible, age-friendly physical activity interventions that promote functional fitness and social interaction. The WHO report Framework for Action on Ageing and Health (2) stresses the importance of enabling older adults to maintain functional ability through supportive environments and targeted interventions. It highlights that accessible technologies can empower older adults to stay active and engaged, improving their quality of life.

The Article Collection *User-Centered Technology for Exercise Optimization in Older Adults* showcases six studies that apply a variety of approaches, from wearable devices and virtual reality environments to artificial intelligence-driven personalization and community-based initiatives. One prominent trend is the integration of augmented reality (AR) and mixed-reality technologies in physical activity and exercise programs.

## Articles in this research topic

Ferreira et al. showed that AR-enhanced multimodal training significantly improved outcomes such as lower limb strength, balance, and cardiorespiratory fitness, outperforming traditional training programs. However, the high cost and complexity of these technologies highlight the need for diverse strategies to promote physical activity. While AR offers immediate physical benefits, its influence on long-term behavioral change remains uncertain. Beyond AR, home-based exergaming and dual-task interventions have proven effective in addressing both physical and cognitive decline (3).

Glatt et al. introduced the Fitbrain program, which combines dual-task and exergaming activities to engage older adults in cognitive and physical challenges. They emphasized that integrating mental and physical exercises can yield synergistic benefits for brain and body function. However, they highlighted challenges in scaling these interventions, including high costs and limited research comparing multimodal to singular approaches. This calls for systematic evaluations to identify the most effective strategies for diverse populations. Remote delivery methods, such as online dance programs, provide additional opportunities to engage older adults.

Hansen et al. investigated a 12-week online dance training program, finding significant improvements in dynamic postural control and gait speed. Their study demonstrated the feasibility of delivering complex, movement-based interventions via virtual platforms. While static postural stability showed little improvement, the gains in dynamic measures point to promising applications for fall prevention. These findings also underscore the potential of remote programs to improve accessibility enabling older adults with geographic, logistical, or mobility barriers to engage in structured physical activity.

The potential of motor-cognitive exergames in rehabilitation settings is further highlighted by Huber et al., who developed the PEMOCS concept to enhance cognitive functioning and gait in chronic stroke survivors. Grounded in Gentile's Taxonomy for Motor Learning, their program introduces a structured, personalized progression that integrates physical and cognitive training. By extending the taxonomy with a third cognitive dimension, they created a framework addressing the complex needs of stroke survivors. Their emphasis on objective performance metrics underscores the importance of data-driven adaptation in optimise intervention efficacy.

Preventive strategies targeting pre-frailty also play a vital role in the spectrum of interventions. In their work, Li et al. introduced PF-Life, a mobile health-supported multicomponent exercise program aimed at reversing pre-frailty in older adults. Their study assessed effectiveness using physical performance measures, body composition analysis, and inflammatory biomarkers, emphasizing the value of early frailty intervention. PF-Life integrates lifestyle components with long-term follow-up, offering a personalized, scalable, and holistic approach to preventing frailty progression, with significant public health implications.

In resource-limiting settings, Dino et al. proposed mixed-reality interventions to promote health among older adults. Combining live coaching and video-based exercises with mixed reality technology, their program employs human-centered design and has cultural relevance. Tailored to the needs of community-dwelling older adults in the Philippines, their protocol highlights the importance of adapting technological solutions to local contexts. A mixed-methods approach, including usability evaluations and participant modelling, provides a comprehensive framework for assessing both effectiveness and user experience.

Collectively, these studies highlight key factors influencing the success or limitations of health interventions for older adults. Technology integration emerges as a recurring theme, enhancing engagement, personalization, and scalability. From AR and exergaming to mixed-reality coaching, technology creates opportunities to make interventions more interactive, adaptable, and appealing. However, as noted by Ferreira et al. and Glatt et al., technology must align with evidence-based frameworks to ensure its effectiveness. Personalization also emerges as a critical component across the studies. Personalization also proves critical, as demonstrated by structured progression in the PEMOCS concept (Huber et al.), user-centered design in mixed-reality programs (Dino et al.), or tailored exercise regimens in PF-Life (Li et al.).

Addressing motivational factors, adherence, and independent activity beyond structured sessions remains a significant challenge. Incorporating gamification, social support, and ongoing feedback may offer promising strategies to bridge this gap. Lastly, the studies also highlight the importance of equity and accessibility in designing and implementing interventions.

## Conclusion

The contributions to this Research Topic collectively highlight the transformative potential of user-centered technologies in optimizing physical activity for older adults. By addressing barriers such as digital literacy, affordability, and accessibility, these studies lay the groundwork for inclusive solutions that support healthy aging. As the global population ages, fostering collaboration among technologists, healthcare providers, and policymakers will be crucial to scaling these innovations effectively.
